# Effectiveness analysis of an internet-based intervention for overweight adolescents: next steps for researchers and clinicians

**DOI:** 10.1186/s40608-016-0094-4

**Published:** 2016-03-09

**Authors:** Helena Fonseca, Ana Prioste, Pedro Sousa, Pedro Gaspar, Maria do Céu Machado

**Affiliations:** Department of Pediatrics, Faculdade de Medicina, Hospital de Santa Maria, Universidade de Lisboa, Av. Prof Egas Moniz 1, 1649-028 Lisboa, Portugal; Rheumatology Research Unit, Instituto de Medicina Molecular, Faculdade de Medicina, Universidade de Lisboa, Lisboa, Portugal; Faculdade de Psicologia, Universidade de Lisboa, Lisboa, Portugal; Escola de Psicologia e Ciências da Vida, COPELABS, Universidade Lusófona de Humanidades e Tecnologias, Lisboa, Portugal; Instituto Politécnico de Leiria, Leiria, Portugal

**Keywords:** Overweight, Internet-based weight management programs, Adolescence, Effectiveness

## Abstract

**Backgrounds:**

The development of effective strategies for the management of overweight in adolescence is a well recognized need. The current study investigates the effectiveness of an e-therapeutic platform (Next.Step) which aims to promote weight management skills and the adoption of health-promoting behaviours among overweight adolescents.

**Methods:**

We conducted a randomized clinical trial with a sample of 80 adolescents. The control group followed the standard intervention. The experimental group was invited to access the platform during 12 weeks in addition to the standard intervention.

**Results:**

Although there was no change in the primary outcomes (body mass index and percentage of fat mass), the results suggest that the program is associated with an improvement in the ‘positive perspective of life’ and ‘benefits perceived from the intervention’, which have been identified as relevant factors for an effective weight management.

**Conclusions:**

Our findings provide little support for the effectiveness of internet-based weight management programs as an add-on to the standard intervention.

**Trial registration:**

NCT01904474

## Background

There is an urgent need for effective interventions that induce behavioural change in overweight adolescents [1]. Successful interventions depend on adherence to treatment, lifestyle change and on the maintenance of the therapeutic support [2, 3]. Several authors highlighted the role of information and communication technologies (ICT) in the management of obesity in adolescence [4, 5]. Internet-based weight management programs have the potential for being an effective option since they allow ongoing contact between the adolescent and the health care provider, which has been associated to a greater adherence to behavioural changes and to an improvement of maintenance of weight lost [6, 7]. Literature has shown that internet-based weight management programs are effective in body mass index (BMI) reduction, physical activity increase [5], increased adherence to behavioural change and maintenance of weight loss [7]. A previous non randomized study with the Next.Step program [8], an e-therapeutic program which aims to promote weight management skills and the adoption of health-promoting lifestyles in overweight adolescents, showed an increase in health responsibility. However, it was mentioned by then that there was need for additional studies in order to understand whether the internet (in general) and the Next.Step (in particular) could be considered as effective communication channels for inducing behavioural change in overweight adolescents [8].

Thus, the current study analyzed the effectiveness of the Next.Step program compared to a standard intervention, in what concerns a set of anthropometric and psychosocial variables. We considered the anthropometric variables (BMI and percentage of fat mass) and quality of life as primary endpoints. Data on health responsibility, positive perspective of life, self-efficacy/adherence to behaviour change and perception of benefits from the intervention (secondary endpoints) were used to evaluate additional effects of the intervention. We hypothesized that the Next.Step program would be more effective on the promotion of weight management than the standard intervention, expecting that anthropometric and psychosocial variables would be positively influenced by the program.

## Methods

### Participants

The sample comprised 80 adolescents (40/40: experimental/control groups), aged between 12 and 18 (*M* = 14.6; *SD* = 1.88), 52.9 % females.

Sample size was calculated according to the power analysis, with BMI, quality of life, treatment adherence and lifestyle used as starting points. We aimed at being able to show differences between the Next.Step participants and the control group with a standardized effect size (Cohen’s d) of 0.4 or larger [9–11]. Assuming a 30 % drop-out rate (in the literature systematic review, the drop-out rate ranged between 4.9 [12] and 30 % [13]), an alpha of 0.05 and a statistical power (1-Beta) of 80 %, we came to a need for recruiting at least 75 adolescents.

### Measures

#### Body Mass Index (BMI) and percentage of fat mass

BMI and percentage of fat mass were measured by trained health professionals from the Paediatric Obesity Clinic (POC), Hospital de Santa Maria, Lisbon, and were extracted from the adolescents’ clinical file. Height was measured to the nearest 0.1 cm, without shoes, with the participant back to the stadiometer, in the Frankfurt position and after an expiratory phase (height stadiometer, SECA 217, Hamburg, Germany). Body weight and body composition (bioelectrical impedance scale InBody 230, Seoul, Korea). Body weight will be measured to the nearest 0.1 kg, in the anthropometric position (with the palms turned into thighs), with the subjects wearing as few clothes as possible, and without shoes or socks. BMI was calculated as body weight in kilograms divided by the square of height in meters [BMI = weight (kg)/height^2^ (m)]. Subjects were classified into overweight (BMI ≥ 85^th^ percentile) according to the age- and sex-specific percentiles proposed by the World Health Organization (WHO).

#### Self-efficacy/adherence behavior and perceived benefits

Adolescents reported on their self-efficacy/adherence behavior to weight control using the Adherence to Weight Control Questionnaire (AWCQ) [14]. The AWCQ is a screening tool that includes 36 items, rated on a 1–5 Likert scale (“Do not agree” to “Totally agree”), organized in two scales: Treatment adherence to weight control and Risk of non-adherence to weight control. The former scale includes four subscales: Self-efficacy/adherence behavior (SEA); Parents and providers’ influence; School and Friends’ influence; and Perceived benefits (PB). In this study, we have only used the SEA and the PB dimensions as we were mostly interested in internal/individual sources of behavioral influence. The SEA dimension includes 12 items such as ‘*I accomplish the treatment even if I’m not in the mood*’. This dimension represents the perception of self-ability and commitment to organize and perform a particular health behavior, i.e., self-confidence in successfully engaging in the weight management program, intention to carry out the treatment and ability to identify the specific strategies to succeed. The PB dimension includes four items (e.g., ‘*I want to lose weight to be healthier*’) related to perceptions of the positive or reinforcing consequences of engaging in the weight management program.

In the previous validation study with a sample of Portuguese adolescents with obesity [14], the SEA and PB dimensions have shown good internal consistency (*α* = .89 and *α* = .77, respectively), similar to the ones found in the current study (*α* = .84; *α* = .75, respectively).

#### Quality of life (QoL)

Adolescents completed the Impact of Weight on Quality of Life-Lite (IWQOL-Lite) [15], which assesses obesity-specific quality of life. The IWQOL-Lite is a 31-item questionnaire scored on five-point Likert scale ranging from 1 (never) to 5 (always). This instrument assesses four dimensions: Body self-esteem; Social life; Physical comfort; and Relation with the family. Engel and colleagues [15] found a good value of internal consistency for the total score. In the current study, we have found a similar value (*α* = .85).

#### Positive life perspective and Health responsibility

Adolescents completed the Adolescent Lifestyle Profile (ALP-R2; adapted and validated for the Portuguese population [16]), which measures the frequency of health promoting behaviors in adolescents. The ALP-R2 includes 36 items rated on a four-point Likert scale ranging from 1 (never) to 4 (always). This instrument assesses seven dimensions: Health responsibility (HR), Physical activity, Nutrition, Interpersonal relationships, Spiritual health, Positive life perspective (PLP), and Stress management. In this study, we have only focused on the PLP and HR dimensions. PLP dimension includes four items (e.g., ‘*I’m excited about the future*’) and HR dimension includes six items (e.g., ‘*I usually ask questions about how to improve my health to my doctor/nurse’*). We opted to focus only on these two dimensions because we were essentially interested in internal/individual sources of behavioral change.

Sousa et al. [16] found satisfactory values of internal consistency for the HR and PLP dimensions. In the current study, we have found good values of internal consistency (*α* = .74 and *α* = .80, respectively).

### Procedures

Participants were selected from the population of adolescents followed at the POC. All eligible adolescents with appointments between 2014 April 1 and 2015 January 31 were included in the study. Adolescents were assigned to the control or experimental groups by alternating patients sequentially. Participants were required to be overweight, aged between 12 and 18, willing to participate in the study and have internet access at least once a week. Exclusion criteria were the presence of severe psychopathology, pregnancy or having been proposed for bariatric surgery. The control group followed the clinical standard intervention, including individual appointments with the paediatrician, dietician and exercise physiologist every 3 months. In addition to the standard intervention, the experimental group was invited to access the e-therapeutic platform, which includes a set of resources, such as educational resources (videos, brochures, menus, weekly tips, access to other links), self-monitoring (food, weight and physical activity records), social support (chats, discussion forums and personalized messages), interactive training and motivational tools (personal goals planning, treatment progression registry, positive reinforcement) [8, 17]. The intervention length was 12 weeks and was based on a case management methodology. So, the program had the direct support of an interdisciplinary team (including paediatrician, nutritionist, exercise physiologist, and psychologist) who intervened when requested by the case manager (nurse). Full presentation of the platform features can be found in detail in the study protocol [17].

The study protocol was approved by the Ethics Committee of the Faculty of Medicine, University of Lisbon. Adolescents and their parents signed an informed consent. The voluntary nature of their participation was explained and confidentiality was assured.

Anthropometric measurements and self-reported questionnaires were collected at 0 months (baseline assessment) and at 3 months (post-intervention assessment).

### Data analysis

All statistical analyses were performed using Statistical Package for Social Science (SPSS) version 22. Descriptive statistics were calculated, and the groups were compared regarding their baseline characteristics by Mann-Whitney *U* test (*U*) for continuous variables and Chi-Square test (*χ*^2^) for nominal variables. To analyze the effectiveness of the Next.Step program compared to the standard intervention, in what concerns a set of anthropometric and psychosocial variables, we conducted multiple repeated-measures factorial ANOVAs. We further performed multiple comparisons using Bonferroni adjustment at *p* < .017, to reduce the probability of type I error. We opted to use this analytic strategy to analyze the two main effects of the factors (time and group) and the interaction effect between them.

## Results

Table 1 presents the descriptive statistics at baseline and baseline characteristics differences between experimental and control groups and the results of the Mann-Whitney *U* test and Chi-Square test. The two groups can be considered comparable and homogeneous in what concerns the sociodemographic, anthropometric and psychosocial variables.

**Table 1:** Descriptive statistics at baseline and differences between the experimental and control groups regarding sociodemographic, anthropometric and psychosocial variables

	Experimental group		Control group		*U*	*p*
	*M*	*SD*	*M*	*SD*		
Age	14.48	1.91	14.52	1.78	718.50	.86
BMI	30.95	6.10	31.42	5.93	765.50	.74
Percentage of fat mass	40.48	6.42	38.81	6.63	791.00	.93
Quality of life	1.65	.59	1.56	.49	732.00	.51
Perceived benefits of the intervention	4.58	.62	4.47	.62	696.50	.30
Positive perspective of life	2.93	.61	3.20	.64	599.00	.05
Self-efficacy/adherence to treatment	3.31	.69	3.30	.69	1602.50	.87
Health responsibility	2.30	.66	2.40	.55	738.50	.55
	*n*	%	*n*	%	*χ* ^2^	*p*
Gender					.45	.50
Male	18	45	21	52.5		
Female	22	55	19	47.5		

We conducted 2 (Time: 0 months vs. 3 months) X 2 (Group: experimental vs. control) repeated measures factorial ANOVAs regarding BMI, percentage of fat mass, QoL, HR, PB, PLP and SEA to analyze the effectiveness of Next.Step compared to the standard intervention. Overall, the 2 X 2 repeated measures factorial ANOVAs regarding BMI, percentage of fat mass, QoL, HR and self-efficacy indicated: a non significant main effect of the time on the scores of the variables; a non significant main effect of the groups on the scores of the variables; and a non significant interaction between the time and the groups on the scores of the variables.

**Fig. 1:**
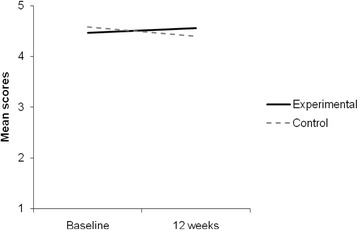
Mean scores of ‘perceived benefits of the intervention’ for experimental and control groups across time (baseline and 12 weeks)

Figure 1 shows the mean scores of perceived benefits of the intervention for the experimental and control groups across time. A 2 X 2 repeated measures factorial ANOVA regarding perceived benefits of the intervention indicated a non significant main effect of the time on the perceived benefits, F(1, 78) = .51, n.s.. Multiple comparisons showed that, in general, the mean score of perceived benefits at 3 months was lower (*M* = 4.52; *SD* = .07) than the mean score at 0 months (*M* = 4.48; *SD* = .06), but there was no significant differences. The main effect of the group on the perceived benefits of the intervention was also non significant, F(1, 78) = .05, n.s. Multiple comparisons showed that the mean score of perceived benefits for experimental group was higher (*M* = 4.49; *SD* = .08) than control mean score (*M* = 4.51; *SD* = .08). Finally, there was a significant interaction between the time and the groups on perceived benefits of the intervention, F(1, 78) = 5.07, *p* < .05, η_p_^2^ = .06, indicating that the allocation in groups had different effects on adolescent’s ratings depending on time. To better understand this interaction, we performed multiple comparisons with Bonferroni adjustment (*p* < .017), comparing each group at 0 months and 3 months. The results showed that, at 0 months, the control group mean score (*M* = 4.58; *SD* = .09) was higher than the experimental group mean score (*M* = 4.47; *SD* = .10); and, at 3 months, the control group mean score (*M* = 4.40; *SD* = .08) was lower than the experimental group mean score (*M* = 4.56; *SD* = .09).

**Fig. 2:**
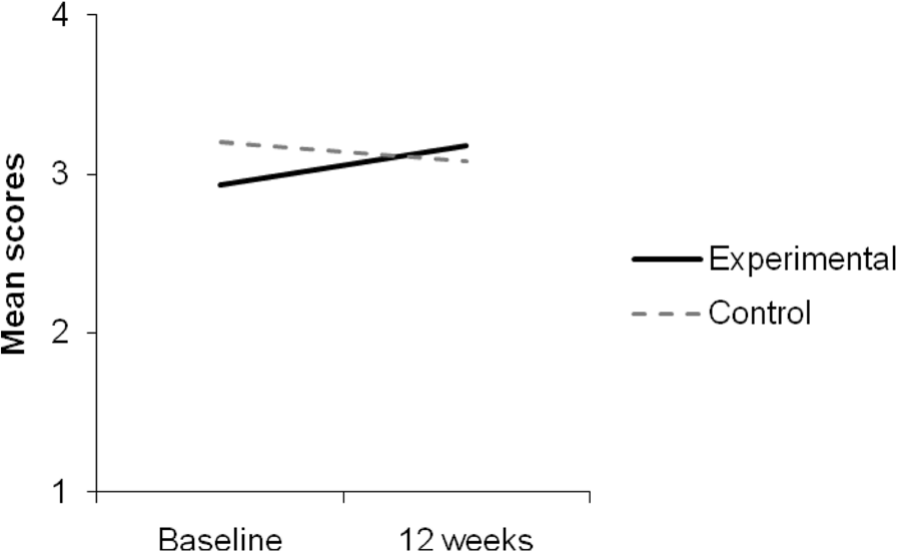
Mean scores of ‘positive perspective of life’ for experimental and control groups across time (baseline and 12 weeks)

Figure 2 shows the mean scores of positive perspective of life for the experimental and control groups across time. A 2 (Time: 0 months vs. 3 months) X 2 (Group: experimental vs. control) repeated measures factorial ANOVA regarding positive perspective of life indicated a non significant main effect of the time on the perceived benefits, F(1, 78) = .92, n.s. Multiple comparisons showed that, in general, the mean score of positive perspective of life at 3 months was higher (*M* = 3.13; *SD* = .07) than the mean score at 0 months (*M* = 3.13; *SD* = .05), but there were no significant differences. The main effect of the group on the positive perspective of life was also non significant, F(1, 78) = .63, n.s. Multiple comparisons showed that the mean score of perceived benefits for the experimental group was lower (*M* = 3.06; *SD* = .07) than the control mean score (*M* = 3.14; *SD* = .08). Finally, there was a significant interaction between the time and the groups on positive perspective of life, F(1, 78) = 7.26, *p* < .01, η_p_^2^ = .09, indicating that the allocation in groups had different effects on adolescent’s ratings depending on time. To better understand this interaction, we performed multiple comparisons with Bonferroni adjustment (*p* < .017), comparing each group at 0 and 3 months. The results showed that, at 0 months, the control group mean score (*M* = 3.20; *SD* = .10) was higher than the experimental group mean score (*M* = 2.93; *SD* = .09); and, at 3 months, the control group mean score (*M* = 3.08; *SD* = .07) was lower than the experimental group mean score (*M* = 3.18; *SD* = .09).

## Discussion

In this study we analyzed the initial effectiveness of an e-therapeutic program (Next.Step) when compared to a standard intervention, regarding the BMI, percentage of fat mass and QoL (primary endpoints), and health responsibility, positive perspective of life, self-efficacy and adherence to behaviour change, and perception of benefits from the intervention (secondary endpoints).

Our results provide little support for the hypothesis that the Next.Step program is more effective than the standard intervention in the promotion of weight management. In fact, the adolescents who joined the Next.Step program did not show any differences regarding the primary endpoints compared to the control group, which supports the results of the preliminary study [8]. Moreover, in contrast with the literature [4, 5], our findings do not suggest that internet-based weight management programs do promote health responsibility, self-efficacy/adherence to behaviour change [8] or quality of life [18]. The lack of significant differences regarding the primary endpoints might be related to the reduced time of intervention. It is also possible that the lack of improvement in the experimental group regarding the identified variables is due to limited exposure to the resources/materials relevant for the intervention, despite the fact that all participants have periodically received both automatic and personalized reminder messages sent through the platform [17]. Furthermore, if a control group without any intervention at all had been used, maybe we would have found more significant differences between the two groups [19].

Despite there was no change in the primary outcomes (body mass index and percentage of fat mass), our findings suggest that the adolescents who joined the Next.Step program showed a significant increase in the indices of positive perspective of life and of perception of benefits of the intervention. Although these variables are secondary outcomes, they have been pointed out as most influential on weight management. For example, a recent qualitative study [20] identified positivity, i.e., the generation of positive emotions, as a relevant feature of a ICT effective weight management program.

This study has several limitations that must be underlined: (i) only one self-reported measure was used to assess each psychosocial variable, so it is questionable whether the findings can be generalized to other measures; (ii) bias associated to self-reported questionnaires (e.g., social desirability); (iii) the absence of a follow-up to evaluate the maintenance of the behavioral change; (iv) the absence of data collection regarding the access frequency to the e-platform.

## Conclusions

In summary, our findings provide little support for the effectiveness of internet-based weight management programs as an add-on to the standard intervention. However, it showed improvement in secondary outcomes that have been identified as relevant for an effective weight management.

For a better understanding of the power of internet-based weight management programs, these findings should be carefully examined in future studies including a wider set of measures and a long-run follow-up.

### Ethics approval and consent to participate

This research involves human participants and an informed consent was signed by all the participants and their parents. The study was approved by the Ethics Committee/Institutional Review Board of the Faculty of Medicine, University of Lisbon, and all procedures were in accordance with the Helsinki declaration and its later amendments.

### Availability of data

The dataset supporting the conclusions of this article is available upon request to the corresponding author.

## Abbreviations

ALP-R2: adolescent lifestyle profile
AWCQ: adherence to weight control questionnaire
BMI: body mass index
HR: health responsibility
IWQOL-Lite: impact of weight on quality of life-lite
PB: perceived benefits
PLP: positive life perspective
POC: paediatric obesity clinic
QoL: quality of life
SEA: self-efficacy/adherence behavior
WHO: World Health Organization
